# Efficacy of flurbiprofen axetil for preventing postanesthetic shivering in patients undergoing gynecologic laparotomy surgeries

**DOI:** 10.1186/s40981-020-00403-x

**Published:** 2020-12-07

**Authors:** Atsushi Kotera

**Affiliations:** Department of Anesthesiology, Kumamoto City Hospital, 4-1-60, Higashimachi, Higashi-ku, Kumamoto City, 862-8505 Japan

**Keywords:** Postanesthetic shivering, Flurbiprofen axetil, Gynecologic laparotomy surgery

## Abstract

**Background:**

Postanesthetic shivering is an unpleasant adverse event in surgical patients. A nonsteroidal anti-inflammatory drug has been reported to be useful in preventing postanesthetic shivering in several previous studies. The aim of this study was to evaluate the efficacy of flurbiprofen axetil being a prodrug of a nonsteroidal anti-inflammatory drug for preventing postanesthetic shivering in patients undergoing gynecologic laparotomy surgeries.

**Method:**

This study is a retrospective observational study. I collected data from patients undergoing gynecologic laparotomy surgeries performed between October 1, 2019, and September 30, 2020, at Kumamoto City Hospital. All the patients were managed with general anesthesia with or without epidural analgesia. The administration of intravenous 50 mg flurbiprofen axetil for postoperative pain control at the end of the surgery was left to the individual anesthesiologist. The patients were divided into two groups: those who had received intravenous flurbiprofen axetil (flurbiprofen group) and those who had not received intravenous flurbiprofen axetil (non-flurbiprofen group), and I compared the frequency of postanesthetic shivering between the two groups. Additionally, the factors presumably associated with postanesthetic shivering were collected from the medical charts. Intergroup differences were assessed with the *χ*^2^ test with Yates’ correlation for continuity category variables. The Student’s *t* test was used to test for differences in continuous variables. Furthermore, a multivariate logistic regression analysis was performed to elucidate the relationship between the administration of flurbiprofen axetil and the incidence of PAS.

**Results:**

I retrospectively examined the cases of 141 patients aged 49 ± 13 (range 21-84) years old. The overall postanesthetic shivering rate was 21.3% (30 of the 141 patients). The frequency of postanesthetic shivering in the flurbiprofen group (*n* = 31) was 6.5%, which was significantly lower than that in the non-flurbiprofen group (*n* = 110), 25.5% (*p* value = 0.022). A multivariate logistic regression analysis showed that administration of flurbiprofen axetil was independently associated with a reduced incidence of postanesthetic shivering (odds ratio 0.12; 95% confidence interval, 0.02-0.66, *p* value = 0.015).

**Conclusions:**

My result suggests that intraoperative 50 mg flurbiprofen axetil administration for postoperative pain control is useful to prevent postanesthetic shivering in patients undergoing gynecologic laparotomy surgeries.

## Background

In the operating room, both an anesthetics-induced thermoregulatory impairment and a cool environment can make a surgical patient hypothermia. Postanesthetic shivering (PAS) is usually triggered by hypothermia and is a stressful complication. According to some previous reports, PAS incidence ranged from 20 to 70% [1–3], and the overall PAS incidence in a meta-analysis was 34% [4]. PAS should be prevented because it triggers numerous adverse effects such as infection, bleeding, delayed wound healing, and myocardial ischemia, leading to prolonged hospital stay [5].

Parecoxib, an elective cyclooxygenase (COX)-2 inhibitor, is effective for preventing PAS [6]. Flurbiprofen axetil is a non-selective cyclooxygenase inhibitor widely used for postoperative pain control, suggesting that it is also useful for preventing PAS through anti-inflammatory effects.

In the present study, I aimed to evaluate the efficacy of intraoperative flurbiprofen axetil administration for preventing PAS in patients undergoing gynecologic laparotomy surgeries.

## Patients and methods

### Patients

The approval for this retrospective observational study (approval no. 567) was provided by the Ethical Committee of Kumamoto City Hospital, Kumamoto, Japan, on October 8, 2020. Patients who underwent gynecologic laparotomy surgeries under general anesthesia with or without epidural analgesia performed between October 1, 2019, and September 30, 2020, were eligible for this study. Patients’ exclusion criteria were as follows: age of < 20, chronic kidney disease with hemodialysis, and postoperative mechanical ventilation following massive intraoperative bleeding. All data were pre-existing data obtained from the patient’s medical records and did not include any personal information that would identify any of the patients. Informed consent from the patients was therefore waived, based on the Ethical Guidelines for Epidemiological Studies issued by the Ministry of Health, Labour and Welfare and Ministry of Education, Culture, Sports, Science, and Technology of Japan. I followed the Strengthening the Reporting of Observational Studies in Epidemiology (STROBE) guidelines [7] as a retrospective observational study, and I published the details of this study on the homepage of Kumamoto City Hospital.

### Data collection

Data were collected from the patients’ medical charts and anesthesia records. Intraoperative administration of intravenous 50 mg flurbiprofen axetil for postoperative pain control was left to the individual anesthesiologist. I thus extracted the presence or absence of the flurbiprofen axetil administration from the patients’ anesthesia records. Factors presumably associated with postanesthetic shivering were also extracted from the patients’ medical charts: patient age, body mass index (BMI), underlying co-morbidities, tumor characteristics (benign or malignant), the operation time, the anesthesia time, blood loss, fluid infusion, fentanyl dosage, remifentanil dosage, body temperature, and the presence or absence of epidural analgesia. A patient’s body temperature was measured by a nasopharyngeal thermometer or rectal thermometer continuously. Intraoperatively, active cutaneous warming with forced air blanket and body core warming with heated fluid management were undergone for all the patients.

In this study, I set the presence of PAS as the endpoint. PAS in this study is defined as a detectable fasciculation of the patient’s face, lip, jaw, neck, shoulder, trunk, or extremities, or visible shaking observed within 1 h after the end of the operation. When PAS occurred in the operating room, the anesthesiologist recorded the event in the patient’s anesthesia record. When PAS occurred in the general ward after leaving the operating room, the nurse staff recorded the event in the patient’s medical chart.

I divided the patients into two groups: those who had received intravenous flurbiprofen axetil at the end of surgery (flurbiprofen group) and those who had not received intravenous flurbiprofen axetil (non-flurbiprofen group), and I compared the frequency of PAS between the two groups. I also analyzed the other factors presumably associated with PAS between the two groups statistically.

In addition, I divided the patients into two groups: those who suffered from PAS (PAS group) and those who did not suffer from PAS (non-PAS group), and a multivariate logistic regression model was used to validate the relationship between the administration of flurbiprofen axetil and the incidence of PAS.

### Statistical analysis

All statistical analyses were carried out using the software program Excel Tokei 2012 (Social Survey Research Information, Tokyo). Intergroup differences were assessed with the *χ*^2^ test with Yates’ correlation for continuity category variables. The Student’s *t* test was used to test for differences in continuous variables. A multivariate logistic regression model was used to adjust for potential confounders and odds ratios were showed with 95% confidence intervals. In the multivariate logistic regression analysis, variables in patient characteristics associated with PAS were also included if the significance was *p* < 0.05 in the univariate analysis. Differences of *p* < 0.05 were considered significant. Descriptive data are presented as the mean ± standard deviation (range).

## Results

During the study period, 144 gynecologic laparotomy surgeries were undergone. However, three patients were excluded because of the exclusion criteria. The background of the eligible 141 patients were shown in Table 1, and the overall incidence of PAS was 21.3% (30 of the 141 patients). Among the 30 cases of PAS, eight were observed at the operating room and residual 22 were observed on the way to the general ward or on arriving at the general ward.

**Table 1:** Clinical data of all the eligible patients

Age (year)	49 ± 13 (21-84)
Body mass index (kg/m^2^)	23.7 ± 4.8 (16.0-46.1)
Co-morbidities	
Hypertension	26 (18.4)
Diabetes mellitus	8 (5.7)
Bronchial asthma	19 (13.5)
Malignancy	70 (49.6)
Operation time (min)	143 ± 56 (47-356)
Anesthesia time (min)	206 ± 65 (103-458)
Blood loss (g)	450 ± 550 (5-3519)
Fluid infusion (ml/kg/h)	8.9 ± 3.2 (3.4-16.7)
Fentanyl dosage (μg/kg)	4.4 ± 1.7 (0.9-10.9)
Remifentanil dosage (mg)	2.0 ± 0.9 (0.8-6.0)
Body temperature (°C)	
Highest body temperature	36.8 ± 0.4 (35.8-38.0)
Lowest body temperature	36.3 ± 0.4 (35.1-37.3)
Epidural analgesia	127 (90.1)
Postanesthetic shivering	30 (21.3)

The data are given as patient’s number (%) or the mean ± standard deviation (range)

The frequency of intravenous flurbiprofen axetil administration was 22% (31 of the 141 patients) and I compared the clinical characteristic between the flurbiprofen group and non-flurbiprofen group (Table 2). The incidence of PAS in the flurbiprofen group (*n* = 31) was 6.5%, which was significantly lower than that in the non-flurbiprofen group (*n* = 110), 25.5% (*p* value = 0.022).

**Table 2:** Comparison of patient characteristics between the flurbiprofen group and non-flurbiprofen group

	Flurbiprofen group (*n* = 31)	Non-flurbiprofen group (*n* = 110)	*p* value
Age (year)	48 ± 15 (21-84)	49 ± 13 (24-84)	0.850
Body mass index (kg/m^2^)	25.0 ± 6.1 (16.4-46.1)	23.3 ± 4.3 (16.0-36.0)	0.155
Co-morbidities			
Hypertension	8 (25.8)	18 (16.4)	0.231
Diabetes mellitus	2 (6.5)	6 (5.5)	0.832
Bronchial asthma	2 (6.5)	17 (15.5)	0.195
Malignancy	17 (54.8)	53 (48.2)	0.513
Operation time (min)	140 ± 56 (47-319)	144 ± 56 (55-356)	0.703
Anesthesia time (min)	199 ± 60 (108-381)	207 ± 67 (103-458)	0.429
Blood loss (g)	438 ± 539 (5-2316)	452 ± 556 (6-3519)	0.831
Fluid infusion (ml/kg/h)	9.9 ± 3.7 (5.2-16.5)	8.7 ± 3.0 (3.4-16.7)	0.169
Fentanyl dosage (μg/kg)	4.4 ± 1.9 (1.3-10.9)	4.4 ± 1.7 (0.9-9.8)	0.685
Remifentanil dosage (mg)	1.9 ± 1.0 (0.8-6.0)	2.0 ± 0.9 (0.8-6.0)	0.616
Body temperature (°C)			
Highest body temperature	36.8 ± 0.4 (36.1-38.0)	36.8 ± 0.4 (35.8-37.8)	0.614
Lowest body temperature	36.4 ± 0.4 (35.5-37.2)	36.3 ± 0.4 (35.1-37.3)	0.431
Epidural analgesia	26 (83.9)	101 (91.8)	0.191
Postanesthetic shivering	2 (6.5)	28 (25.5)	0.022

The data are given as patient’s number (%) or the mean ± standard deviation (range)

Univariate analysis of patient characteristics between the PAS group and non-PAS group was shown in Table 3. There were significant differences in age, remifentanil dosage, and administration of flurbiprofen axetil. Age in the PAS group was 41 ± 9 year (range 24-67 year), which was significantly lower than that in the non-PAS group, 51 ± 13 years (range 21-84 year). Remifentanil dosage in the PAS group was 2.4 ± 1.2 mg (range 0.8-6.0 mg), which was significantly higher than that in the non-PAS group, 1.8 ± 0.8 mg (0.8-6.0 mg). The administration of flurbiprofen axetil in the PAS group was 6.7% (2 of the 30 patients), which was significantly lower than that in the non-PAS group, 26.1% (29 of the 111 patients).

**Table 3:** Univariate analysis of patient characteristics between the postanesthetic shivering (PAS) group and non-postanesthetic shivering (non-PAS) group

	PAS group (*n* = 30)	Non-PAS group (*n* = 111)	*p* value
Age (year)	41 ± 9 (24-67)	51 ± 13 (21-84)	< 0.001
Body mass index (kg/m^2^)	23.3 ± 3.9 (17.4-36.0)	23.8 ± 5.0 (16.0-46.1)	0.774
Co-morbidities			
Hypertension	4 (13.3)	22 (19.8)	0.416
Diabetes mellitus	1 (3.3)	7 (6.3)	0.532
Bronchial asthma	4 (3.3)	15 (13.5)	0.195
Malignancy	13 (43.3)	57 (51.3)	0.976
Operation time (min)	166 ± 75 (75-356)	137 ± 49 (47-319)	0.096
Anesthesia time (min)	233 ± 92 (114-458)	198 ± 54 (103-381)	0.103
Blood loss (g)	723 ± 837 (10-3519)	376 ± 418 (5-2316)	0.093
Fluid infusion (ml/kg/h)	8.8 ± 2.9 (3.6-14.2)	9.0 ± 3.2 (3.4-16.7)	0.924
Fentanyl dosage (μg/kg)	4.2 ± 1.8 (1.6-9.8)	4.7 ± 1.7 (0.9-10.9)	0.273
Remifentanil dosage (mg)	2.4 ± 1.2 (0.8-6.0)	1.8 ± 0.8 (0.8-6.0)	0.015
Body temperature (°C)			
Highest body temperature	36.8 ± 0.3 (36.3-37.4)	36.8 ± 0.4 (35.8-38.0)	0.635
Lowest body temperature	36.2 ± 0.5 (35.1-37.0)	36.3 ± 0.4 (35.1-37.3)	0.549
Epidural analgesia	29 (96.7)	98 (88.3)	0.173
Flurbiprofen axetil	2 (6.7)	29 (26.1)	0.022

*PAS* postanesthetic shivering

The data are given as patient’s number (%) or the mean ± standard deviation (range)

Table 4 shows the effects of independent variables from patient characteristics related to PAS with a multivariate logistic regression analysis. This analysis demonstrated that administration of flurbiprofen axetil (odds ratio 0.12; 95% confidence interval, 0.02-0.66) as well as age (odds ratio 0.92; 95% confidence interval, 0.87-0.96) and remifentanil dosage (odds ratio 2.00; 95% confidence interval, 1.24-3.23) was independently associated with PAS.

**Table 4:** Multivariate logistic regression analysis of patient characteristics associated with the incidence of postanesthetic shivering (PAS)

Variable	Odds ratio (95% confidence interval)	*p* value
Age	0.92 (0.87-0.96)	< 0.001
Remifentanil dosage	2.00 (1.24-3.23)	0.005
Flurbiprofen axetil	0.12 (0.02-0.66)	0.015

## Discussion

PAS is a common postoperative complication during the recovery term after general anesthesia, but there is still no clear consensus about the best strategy for its prophylactic [8]. My present retrospective findings indicate that flurbiprofen axetil being a prodrug of NSAID is effective for preventing PAS in patients undergoing gynecologic laparotomy surgeries.

Intraoperatively, the surgical stress produces various cytokines and those induce the COX-2 expression. The COX-2 plays an important role in the PGE_2_ synthesis from the arachidonic acid, and the PGE_2_ raises a set point for body temperature control [9]. Then, in a postanesthetic patient, shivering can occur easily until body temperature will reach its new raised set point not only in a hypothermic patient but also in a normothermic patient [3]. Therefore, it is considered that a COX-2 inhibitor is useful for preventing the incidence of PAS. It has been reported that PAS in the parecoxib administration group was 5.0% (90 cases of 1806 patients), which was significantly lower than that in the non-parecoxib administration group at 13.3% (230 cases of 1719 patients) (odds ratio = 0.21, 95% confidence interval = 0.16-0.29) [10].

Flurbiprofen axetil is an injectable non-selective cyclooxygenase inhibitor and a prodrug of NSAID. It can reduce the systematic levels of proinflammatory cytokines and inhibit the synthesis of PGE_2_ [11]. Thus, I had a hypothesis that flurbiprofen axetil could prevent PAS as well as a parecoxib. Actually, it has been reported that, in the patients undergoing a video-assisted thoracic surgery, the incidence of PAS in the flurbiprofen group was significantly lower than that in the non-flurbiprofen group [3.1% (1/32) vs. 24.6% (28/114), *p* value = <0.01] [12]. In the present study, intraoperative administration of flurbiprofen axetil was also effective to prevent PAS, and I found that flurbiprofen axetil was independently associated with a reduced incidence of PAS as well as a parecoxib. Flurbiprofen axetil has the benefits of fast absorption, high availability, and high affinity to the surgical site and inflammatory tissues [13]. Injected flurbiprofen axetil is rapidly converted to its activated form, flurbiprofen, by esterase in plasma, and its plasma concentration shows a maximum level within 5 min after the administration [14]. I thus consider that intravenous single shot of flurbiprofen axetil at the end of surgery in my present study is sufficient to prevent the incidence of PAS, and its effect would continue for several hours in the postanesthetic period.

The appropriate dose of flurbiprofen axetil in order to prevent PAS is unclear. In the previous report, single shot of 1 mg/kg flurbiprofen axetil was administered for postoperative pain management [15]. I did not verify the dosage of flurbiprofen axetil according to a patient’s body weight. I injected 50 mg flurbiprofen axetil to all the patients’ series based on the recommended dose for an adult patient. Fortunately, in my present study, there were no serious adverse effects such as postoperative bleeding due to platelet dysfunction, gastrointestinal ulcer or bleeding, and acute kidney dysfunction.

My study has several limitations. First, the sample size of patients was relatively small, and the data reflect outcomes at a single center. My findings may thus not be generalizable for patients undergoing gynecologic laparotomy surgery as a whole. Further studies with larger number of patients at multiple centers are needed to test my findings. Second, my findings were limited in gynecologic laparotomy surgery. In general, a female patient shows a higher incidence of shivering than a male patient [16]. I thus consider that my findings would be informative for many anesthesiologists; however, further studies with other type of surgeries are needed to validate our findings. Third, this study was a retrospective investigation. I cannot exclude the possibility of important differences in confounders associated with the relationship between flurbiprofen axetil and PAS even after a multivariate logistic regression analysis because of the possible selection bias in a retrospective observational study design. As the results in this study, remifentanil has been reported to be independently associated with an increased incidence of PAS (odds ratio = 2.17, 95% confidence interval = 1.76-2.68) [17]. Prospective studies are thus needed to validate my findings in the future.

## Conclusion

My result suggests that intraoperative 50 mg flurbiprofen axetil administration for postoperative pain control is useful for preventing postanesthetic shivering in patients undergoing gynecologic laparotomy surgeries.

## Data Availability

The datasets analyzed during this study are available from the corresponding author on reasonable request.

## Abbreviations

PAS: Postanesthetic shivering
COX-2: Cyclooxygenase-2
NSAID: Nonsteroidal anti-inflammatory drug
BMI: Body mass index
PGE_2_: Prostaglandin E_2_
